# Synergistic advances in natural fibre composites: a comprehensive review of the eco-friendly bio-composite development, its characterization and diverse applications

**DOI:** 10.1039/d4ra00149d

**Published:** 2024-05-31

**Authors:** Santhosh Nagaraja, Praveena Bindiganavile Anand, Mohan Kumar K., Muhammad Imam Ammarullah

**Affiliations:** a Department of Mechanical Engineering, MVJ College of Engineering Bangalore 560067 Karnataka India; b Department of Mechanical Engineering, Nitte Meenakshi Institute of Technology Bangalore 560064 Karnataka India; c Department of Mechanical Engineering, Faculty of Engineering, Universitas Diponegoro Semarang 50275 Central Java Indonesia imamammarullah@gmail.com; d Undip Biomechanics Engineering & Research Centre (UBM-ERC), Universitas Diponegoro Semarang 50275 Central Java Indonesia; e Biomechanics and Biomedics Engineering Research Centre, Universitas Pasundan Bandung 40153 West Java Indonesia

## Abstract

In recent years, there has been enhanced interest in the domain of natural fibre composites (NFCs) because of their capacity to provide eco-compatible solutions to ever-increasing ecological concerns. This review provides an intensive assessment of the current situation with examination, progress, and applications concerning NFCs. Natural fibres, *viz.*, jute, kenaf, ramie, banana, coir, wheat grass, *etc.*, and their scope in the development of sustainable composites, techniques involved in the fabrication of the composites, characterization techniques, *viz.*, thermo-mechanical and morphological, biodegradability, dampness retention attributes, and potential applications have been extensively reviewed and reported. Besides, this review encompasses the deterrents and conceivable outcomes connected to NFCs, alongside their environmental implications and monetary feasibility. Through a critical evaluation of the existing literature, this article provides a detailed summary of NFCs for real-time engineering applications. It also provides insights into sustainability practices through NFCs.

## Abstract 2

Through a critical evaluation of the existing literature, this article provides a detailed summary of NFCs for real-time engineering applications. It also provides insights into sustainability practices through NFCs.

## Graphical abstract

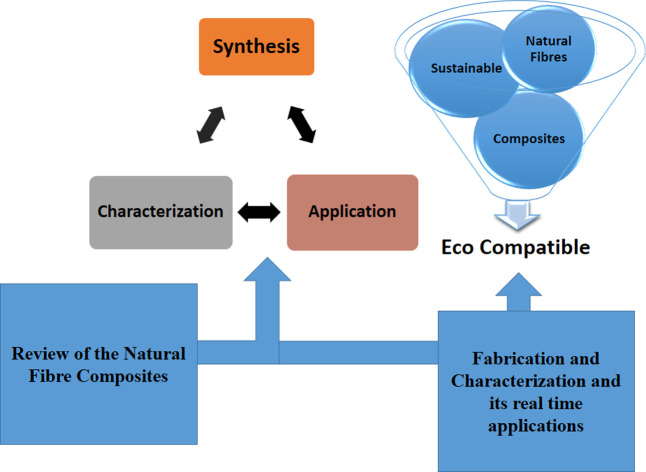

## 1. Introduction

NFCs have seen a noticeable upsurge in popularity recently, mostly because of their promise to be a sustainable option in material engineering.^1^ Finding materials that reduce carbon emissions, improve resource efficiency, limit ecological effects, and are ecologically benign has become increasingly important due to growing environmental concerns and the need for sustainability. Because they combine the benefits of natural fibres with the versatility of matrix materials, NFCs are starting to look like a competitive alternative.^2^

The objective of this study is to conduct a thorough analysis of NFCs and their significant contribution to enhancing sustainability within the field of material engineering. This study aims to shed light on the potential of NFCs in addressing environmental issues and advancing sustainable practices by carefully examining the state-of-the-art research and accomplishments in this field. There are several advantages to using natural fibres in composites. These fibres are sustainable resources that reduce dependency on non-renewable alternatives because they are obtained from botanical, zoological, and cellulose-based sources.^3,4^ Additionally, they are a very desirable option for reinforcing agents in composite materials due to their inherent qualities, which include remarkable strength-to-weight ratio, low mass, and organic biodegradability.^5,6^

Enhancing the characteristics of NFCs is largely dependent on the processing techniques used in their creation.^7^ The strategies related to the extraction, processing, and modification of fibres are intended to increase the mechanical efficiency and overall sustainability of materials by improving the compatibility between natural fibres and matrices.^8^ The reinforcement techniques include fibres that are randomly oriented, fibres that are aligned, and hybrid composites,^9^ each of which is designed to change the mechanical characteristics of the composite to meet the needs of a particular application. Through the skilful blending of multiple fibre types, scientists and engineers can achieve an advantageous blend of strength, stiffness, and other desired characteristics.^10^ A detailed evaluation of mechanical properties such as tensile and flexural strength, impact resistance, thermal conductivity, water absorption, and dimensional stability is necessary to determine their appropriateness for various industries.^11^

The automotive, construction, packaging, and aerospace industries are among those that have embraced the use of NFCs to a large extent. It is feasible to reduce adverse environmental consequences, boost energy efficiency, and support sustainability by incorporating these composites.^12,13^ However, there are still barriers that prevent NFCs from being widely used. The difficulties include the fibres' accessibility, susceptibility to moisture, need for standardization and quality control, market acceptance, and affordability.^14,15^ It is imperative to aggressively pursue ongoing research, develop technologies, and foster collaboration among academics, business leaders, and legislators to overcome these obstacles.^16,17^

This review study provides a comprehensive and in-depth analysis of NFCs, covering their unique properties, manufacturing processes, applications, and sustainability factors. By synthesizing existing information and identifying potential research fields, this review aims to improve the integration of NFCs into material engineering.

### 1.1 Background and motivation

The significance of environmental sustainability and the urgent need to lessen the harm that different industries inflict on the environment have received significant attention in recent years.^18^ Traditional industrial materials, such as metals and synthetic polymers, have several disadvantages, including non-renewability, waste production, and carbon emissions.^19^ This emphasizes how important it is to research and develop substitutes that adhere to environmental and sustainable standards.

NFCs have garnered interest as a potential solution for these issues.^20^ Natural fibres from plants, animals, and cellulose-based materials are incorporated into a matrix to create these composites.^21^ These composites offer enhanced mechanical performance and a decreased environmental impact by fusing the unique properties of natural fibres with the flexibility and processability of matrix materials.^22^

The regenerative and sustainable qualities of NFCs lend encouragement to their use.^23^ These biodegradable fibres offer a green solution to the problems traditional materials often create when it comes to garbage collection and disposal since they come from sustainable sources such as agricultural waste, flax, hemp, jute, and bamboo.^24–26^

NFCs also provide several benefits compared to conventional materials.^27^ Natural fibres are valued in a wide range of industries, including consumer goods, construction, automotive, and packaging, due to their high specific strength, stiffness, low density, and remarkable thermal insulation capabilities.^28,29^

NFCs have the significant advantage of requiring less energy for their manufacture than conventional materials, meaning that carbon emissions are reduced.^30,31^ This aligns with international initiatives to mitigate climate change and promote ecologically sustainable production methods.^32,33^ This review provides a thorough analysis of NFCs and their pivotal function in promoting sustainability in the field of material engineering.^34^ Examining the characteristics, production processes, uses, and sustainability of these composites will increase the public knowledge and acceptance of these materials as workable and sustainable substitutes.^35^

### 1.2 Objectives of the review

• Examine and sort various natural fibre types for composites: the purpose of this review is to examine and sort the various natural fibre types that are frequently used for composite materials. It looks at cellulose-based fibres (like bamboo and wood), animal-based fibres (like wool and silk), and plant-based fibres (like jute, hemp, and flax). If scientists and engineers have a solid grasp of the unique characteristics and qualities of different natural fibres, they will be able to reliably choose fibres for specific applications.

• Natural fibre processing methods: several methods of sourcing, processing, and altering natural fibres for inclusion in composite materials are discussed in this review. Techniques including retting, decortication, chemical treatment, and mechanical treatment will all be covered. These techniques remove contaminants, improve fibre performance, and promote compatibility between the matrix and the fibre. Knowing these methods will help with understanding the measurements and factors that must be considered to obtain the necessary fibre characteristics.

• Investigate techniques for strengthening NFCs: this review investigates many techniques employed to enhance the mechanical characteristics of NFCs. It provides great detail on how to use randomly orientated, hybridized, or aligned fibres to build composites. By investigating how factors like volume ratio, fibre alignment, and hybridization affect composite behaviour, scientists can create methods to achieve better mechanical properties to meet the requirements for specific applications.

• Analyze NFC's mechanical properties: the mechanical properties of NFCs, including tensile strength, flexural strength, impact resistance, hardness, and fatigue performance, are covered in detail in this study. A thorough analysis of previous research and experimental data provides a comprehensive understanding of the ways variables like fibre type, matrix material, processing parameters, and interactions between the fibre and matrix affect the mechanical performance of composites.

• Examine composites with natural fibres for a range of uses: the various uses of NFCs in a variety of industries are investigated, such as consumer products, automotive, construction, aerospace, and packaging. Using lessons from successful case studies and real-world applications, the essay aims to illustrate the versatility and promise of NFCs as more environmentally responsible substitutes for traditional materials.

• Identify potential and difficulties: the potential and problems associated with the integration of composites comprised of natural fibres are identified and explored. This includes elements like the availability of fibre, moisture sensitivity, standardization, quality assurance, market acceptance, and pricing. Understanding these difficulties can help academics and professionals in the field create plans to overcome barriers and seize the chances that natural fibre composite materials provide.

• Evaluate the sustainability and ecological impact of NFCs: we examine things like the capacity of natural fibres for renewable energy, their biodegradability, the life cycle assessment of composite materials, and their carbon footprint. The review illustrates the value of NFCs in environmentally conscious material engineering techniques through this analysis.

**Fig. 1:**
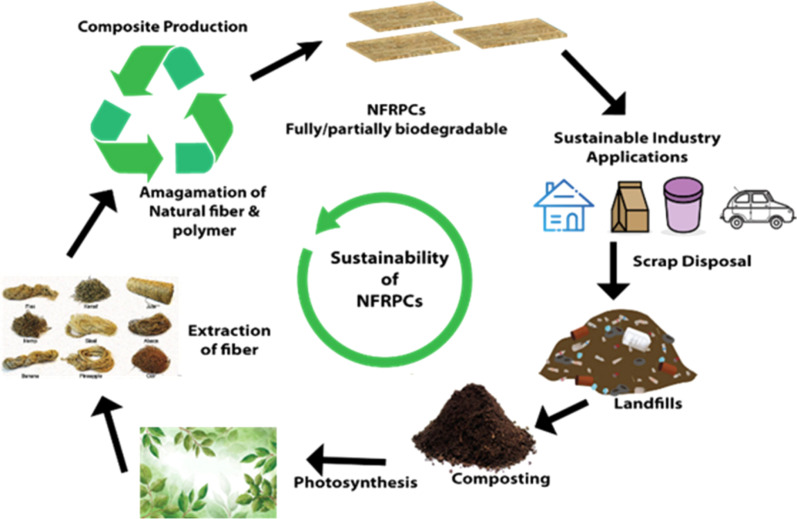
A schematic block diagram of the sustainability of Natural Fibre Reinforced Polymer Composites. Adopted from ref. 36 with permission. Copyright 2022 MDPI *Polymers* [Open Access Creative Commons CC-BY-NC 4.0 license].

The sustainability of composites reinforced with natural fibres and polymers is depicted in Fig. 1. The objectives of this review roughly align with the conceptual block diagram that was created using S. H. Kamarudin's article.^36^

## 2. Categories of wood and non-wood natural fibres

Characterizing NFCs involves considering both wood and non-wood natural fibres, each with unique properties and applications. Wood fibres, derived from various sources such as softwoods (*e.g.*, pine, spruce) or hardwoods (*e.g.*, oak, maple), offer excellent mechanical properties, including high tensile and flexural strength. They also possess inherent durability and resistance to decay, making them ideal for outdoor applications like decking, fencing, and construction. Non-wood natural fibres, on the other hand, encompass a diverse range of materials such as jute, hemp, flax, sisal, and kenaf. These fibres offer advantages such as lower density, biodegradability, and renewable sourcing, making them environmentally friendly alternatives to synthetic fibres. Non-wood fibres are commonly used in automotive interiors, packaging materials, and consumer goods due to their lightweight nature and good impact resistance. However, both wood and non-wood fibres require careful processing and treatment to ensure proper adhesion with the polymer matrix and to mitigate moisture absorption, which can lead to dimensional instability and degradation of mechanical properties over time. Overall, understanding the properties and characteristics of both wood and non-wood natural fibres is essential for designing NFRCs tailored to specific applications, balancing factors like performance, cost, and sustainability.

**Fig. 2:**
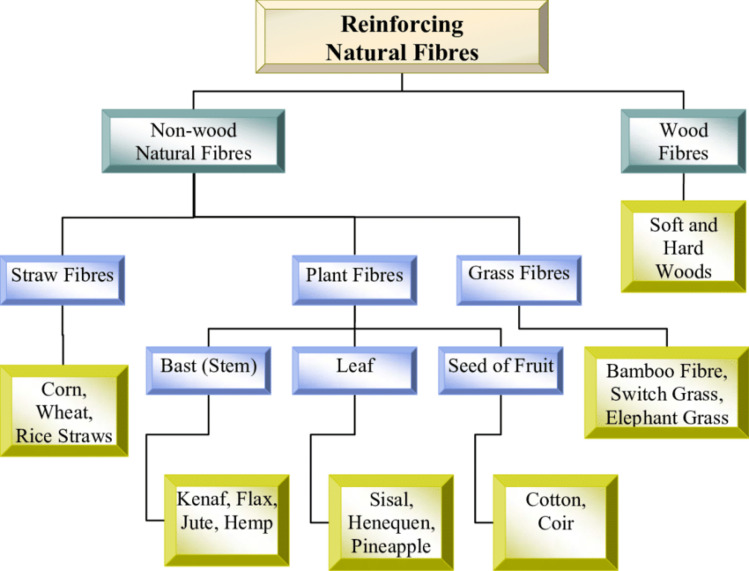
Categories of natural fibres. Adopted from ref. 37 with permission. Copyright Mokhtar, Munirah *et al.*, Universiti Teknologi Malaysia, 2005.

Researchers and engineers are increasingly attracted to natural fibre-reinforced polymeric materials as alternatives to traditional fibres. Fig. 2 gives the detailed classification of natural fibres.^37^ This shift is driven by the numerous advantages attributed to natural fibres (NFs), which encompass their biodegradability, eco-friendliness, affordability, and lightweight nature.

## 3. Processing techniques

### 3.1 Fibre extraction and preparation

Fibre extraction and preparation is a major aspect that needs to be carefully studied. The first step in using natural fibres for composite materials is to prepare and extract the fibres.^38^ Depending on the fibre type and its source material, several extraction techniques are used. For plant-based fibres, the fibres are extracted from the plant stalks or leaves using techniques like retting, decortication, and mechanical separation.^39^ Shearing or reeling is the method used to obtain the fibres from animal-based materials like silk or wool.^40^ Regarding cellulose-based fibres such as cotton, ginning or chemical treatments may be required to remove impurities and make the fibres appropriate for use in composite materials.^41^ To guarantee the quality, purity, and homogeneity of the fibres inside the composite material, it is imperative to follow the proper procedures for fibre extraction and preparation. Table 1 gives the different fibre extraction methods and their examples.^42^

**Table 1:** Different fibre extraction methods. Adopted from ref. 42 with permission. Copyright 2016, Springer

Type of technique	Extraction method	Examples
Physical and mechanical	(a) Grinding	Disc mill, centrifugal mill
	(b) Micronization	Ball mill, jet mill, high-pressure microfluidization
Retting	(a) Water	Hot water extraction, dew extraction
	(b) Chemical	Alkaline, acidic treatment
	(c) Enzymatic	Cellulases, xylanases, laccases, pectinases, *etc.*

### 3.2 Fibre modification and surface treatment

**Fig. 3:**
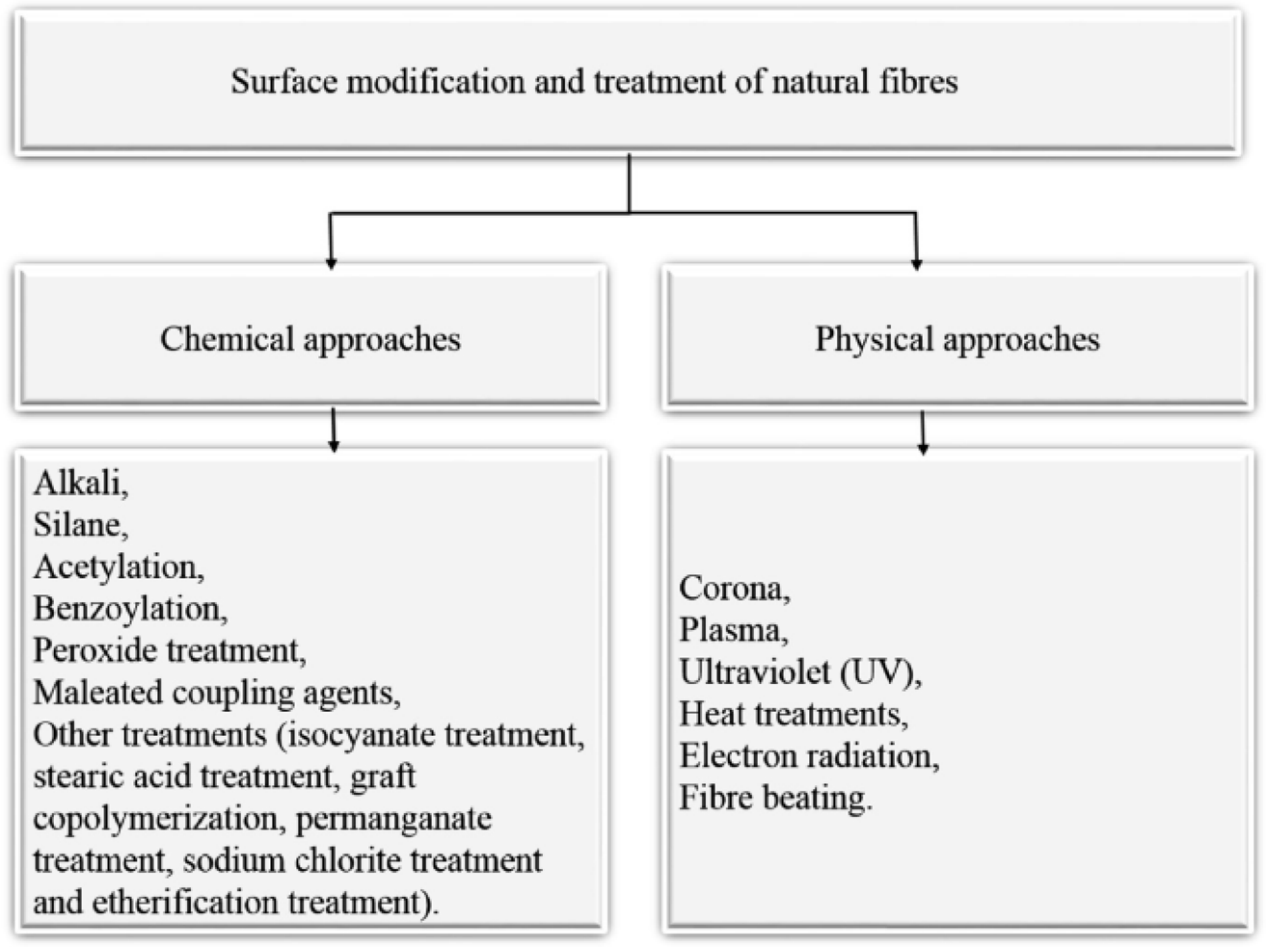
Schematic of different natural fibre modification and surface treatment techniques adopted from ref. 48 with permission. Copyright 2021, Springer.

**Fig. 4:**
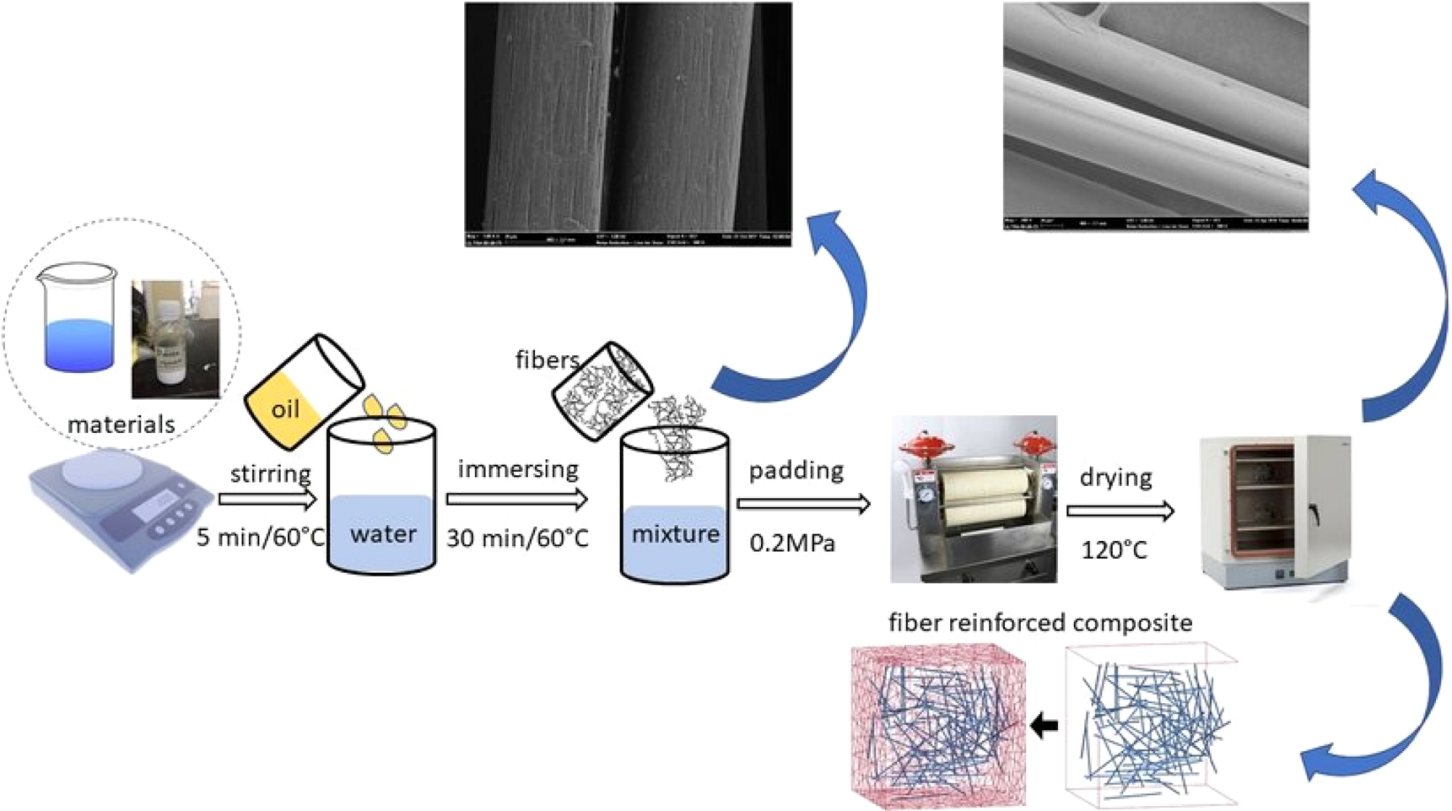
Steps involved in the surface treatment and modification of natural fibres adopted from ref. 49 with permission. Copyright 2019, Elsevier.

Fibre modification and surface treatment are vital to improve their mechanical properties and their compatibility with the matrix material.^43^ To achieve the appropriate fibre lengths, these fibre alterations may include physical procedures like cutting, chopping, or grinding.^44^ Chemical treatments, including silane or alkali treatments, are frequently used to increase moisture resistance, strengthen fibre-matrix bonding, and remove contaminants.^45,46^ These treatments result in changes to the surface characteristics of the fibre, such as its chemical makeup and roughness, which promote better bonding and increased interfacial adhesion between the fibre and the matrix.^47^ Fibre modification and surface treatment techniques are critical for optimizing the mechanical performance and longevity of NFCs. Fig. 3 presents a schematic representation of different physical and chemical methods for surface treatment and natural fibre modification to improve the properties for use in the creation of sustainable composites.^48^Fig. 4 illustrates the procedures used for surface treatment and fibre modification.^49^

### 3.3 Matrix materials and formulation

The matrix material in NFCs acts as a binder for the fibres and transfers applied loads.^50^ Many matrix materials can be used, including thermoplastic polymers (polypropylene, polyethylene, polylactic acid) and thermoset polymers (epoxy, polyester, phenolic resins).^51^ The choice of matrix material depends on the integrated properties that are needed in the mechanical, thermal, and environmental domains. To maximize fibre compatibility, support mechanical efficacy, and customize specific features, the matrix formulation involves the prudent addition of additives such as coupling agents, plasticizers, and fillers.^52^ To achieve a homogeneous dispersion of fibres inside the matrix material, this formulation process additionally makes use of blending and processing techniques, including melt mixing, solution casting, and compression moulding.^53^

Effective processing methods play a critical role in maintaining the performance characteristics of composites made of natural fibres.^54^ To ensure high-quality fibres with the right qualities, it is essential to use the right methods for fibre extraction, preparation, modification, and surface treatment.^55^ Moreover, the overall characteristics and usefulness of the composite are largely determined by the formulation and choice of matrix materials.^56^ The control and improvement of these processing techniques are essential prerequisites for obtaining cohesive and uniform NFCs with enhanced mechanical properties and increased sustainability.^57^

## 4. Reinforcement methods

### 4.1 Randomly oriented fibre composites

Composites featuring randomly oriented fibres are defined by fibres distributed without any specific arrangement within the matrix material.^58^ Typically, these fibres are chopped or maintained at shorter lengths, and they are randomly distributed as part of the composite fabrication procedure. This reinforcement technique is frequently adopted for applications necessitating cost-effectiveness and mass production.^59^ Randomly oriented fibres bestow isotropic mechanical attributes, signifying that the composite displays uniform properties irrespective of the direction.^60^ These randomly oriented fibres enhance the composite's strength, stiffness, and capacity to withstand impact, rendering it well-suited for scenarios where directional characteristics hold less significance.^61^

### 4.2 Aligned fibre composites

In aligned fibre composites, the fibres are arranged inside the matrix material in a particular direction. This is accomplished by employing techniques such as filament winding, unidirectional fibre cassettes, and carefully laying continuous fibres in a specific alignment.^62^ The mechanical properties of aligned fibre composites are improved in the aligned direction, resulting in higher tensile and flexural strength. Engineers may carefully tune the properties of the composite to satisfy the needs of specific loads with the help of this reinforcement approach.^63^ Aligned fibre composites are widely used in applications where directional strength and stiffness are critical, including components needing extraordinary performance, sports equipment, and aircraft constructions.^64^

### 4.3 Hybrid composites

Hybrid composites combine reinforcements or several types of fibres into a single matrix material.^65^ By utilizing the unique qualities of each type of fibre, this approach aims to produce a composite material with improved performance. Combining fibres that are randomly oriented and aligned with other reinforcements, such as nanoparticles or microfillers, is known as hybridization.^66^ The goal is to combine the qualities of several fibre types in a way that produces a cohesive blend of characteristics that includes resilience, impact resistance, stiffness, and strength.^67^ For applications requiring a synthesis of properties, hybrid composites offer the ability to improve composite performance.^68^

The selection of a reinforcement approach hinges on factors such as desired mechanical attributes, financial considerations, processing capabilities, and specific application requisites. Randomly oriented fibre composites have been proven to be suitable for contexts valuing isotropic properties and cost-efficiency.^69^ Aligned fibre composites take precedence when directional strength and stiffness hold paramount importance.^70^ Hybrid composites present the opportunity to derive composites with enhanced mechanical characteristics by fusing diverse fibre types and reinforcements, showcasing adaptability and customizability.^71^

## 5. Characterization of natural fibre-reinforced polymer composites

**Fig. 5:**
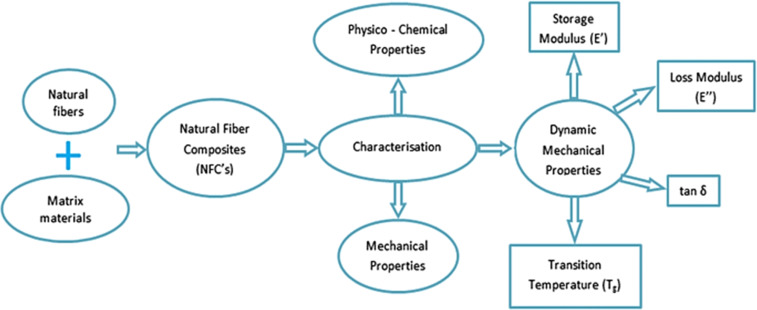
Schematic block diagram of the characterization techniques of NFCs. Adopted from ref. 72 with permission. Copyright Springer, 2019.

The process of characterizing natural fibre-reinforced polymer composites entails the evaluation and comprehension of the properties and responses exhibited by the composite material. This characterization procedure aids in appraising the performance and appropriateness of the composites in relation to particular applications. Fig. 5 is a schematic block diagram of the characterization of natural fibre-reinforced polymer composites.^72^

### 5.1 Mechanical characterization of NFCs

NFCs vary greatly in their mechanical properties. Mechanical properties (*e.g.*, tensile, flexural, and impact) are highly dependent on different factors such as fibre and matrix type, interfacial bonding between the fibre and matrix, fibre dispersion and orientation, and processing, among others. By increasing their mechanical performance, the capabilities and applications of natural-fibre-reinforced composites will be expanded.

One method to increase the mechanical performance of NFCs to broaden their applications is hybridization.^73^ Generally, natural-fibre-reinforced hybrid composites are produced by hybridizing natural fibres with either natural or synthetic fibres with superior properties (*e.g.*, higher mechanical strength, chemical stability, non-toxicity, resistance to high temperatures, and thermal or acoustic insulation). Satyanarayana *et al.*^74^ investigated the influence of hybridisation on the mechanical and thermal properties of intra-laminar natural-fibre-reinforced hybrid composites. They concluded that the mechanical properties are improved *via* the hybridization of sisal-based composites with ramie, sisal, and curauá fibres. On the other hand, they stated that hybridization did not significantly affect the thermal stability of the composites studied.

Fuentealba *et al.*^75^ studied natural-fibre-reinforced composites using 10, 20, and 30% bleached hemp fibres (by weight) in the polyamide 6 polymer matrix structure. They stated that it is possible to obtain tensile strengths higher than glass-fibre-reinforced polyolefin and explained that this effect was due to the strong adhesion between the fibres and the polymer matrix and the good distribution of the fibres in the composite.

It is known that the thermal stability of natural-fibre-reinforced composites is a relevant aspect to be considered as the processing temperature plays a crucial role in the fabrication process. At higher temperatures, the natural fibre components (*e.g.*, cellulose, hemicellulose, and lignin) start to degrade and the major properties (mechanical and thermal) of the composite change. Santhosh *et al.*^76^ presented an overview of the recent advancements made regarding the thermal properties of natural- and hybrid-fibre-reinforced composites in thermoset and thermoplastic polymeric matrices. The methods used to determine the thermal properties of natural and hybrid composites along with the main factors that affect the thermal properties of natural and hybrid fibre composites (fibre and matrix type, the presence of fillers, fibre content and orientation, the treatment of the fibres, and manufacturing process) were discussed. They stated that it is crucial to ensure that the natural fibres used in composites can withstand the heat required during the fabrication process and retain their characteristics.

Datta *et al.*^77^ studied the thermal properties and ballistic performance of kenaf-fibre-reinforced epoxy composites and concluded that the composites reinforced with 30 vol% kenaf fibre presented the best results. Praveena *et al.*^78^ investigated the ballistic behaviour of uni- and bi-directional pineapple leaf fibre (PALF)-reinforced epoxy composites functionalized with graphene oxide (GO). They found that bi-directional GO non-functionalized PALF fibres in a GO-reinforced matrix showed the best results, indicating their possible application as a second layer in multilayered armour systems.

Nishino *et al.*^79^ studied the possibility of improving the mechanical, thermal, and electrical properties of kenaf-fibre-reinforced composites *via* the incorporation of 30% lignin in polypropylene (PP) matrix composites. They found improved thermal stability and higher thermal diffusivity compared to pure PP but the tensile strength decreased. It was concluded that further studies are necessary to improve the adhesion between the PP polymer matrix and kenaf core fibres and lignin.

The main natural fibres studied and used in the industry (*e.g.*, jute, sisal, kenaf, and flax) are well-established on the global market with a well-defined production line. However, new promising types of natural fibres are being discovered and studied. For instance, Nosbi *et al.*^80^ investigated pultruded kenaf fibre, while F. M. Salleh *et al.*^81^ studied the properties of kenaf fibres for possible application as a natural fibre reinforcement in composite materials. Nevertheless, some improvements are needed in their production line to be more commercially affordable and to enable their widespread use.

Both thermoplastic and thermoset polymers are used as matrices in natural-fibre-reinforced polymer composites. There is increased interest in the scientific community regarding the use of bio-based polymers in composites because combining these matrices with natural fibres produces “green composites” or “bio-composites”. Anuar *et al.*^82^ used three types of hybrid matrices based on the Dammar natural hybrid resin and studied the mechanical and chemical properties of flax-fibre-reinforced composite materials, while Deka *et al.*^83^ used bio-based resins to fabricate green composites reinforced with short flax, hemp, and jute fibres. However, the price of these bio-based matrices is still higher than their corresponding petroleum-based counterparts.

Another area currently undergoing rapid development is the application of natural fibres as reinforcements in composites produced *via* additive manufacturing (AM or 3D printing). This technology allows for the fabrication of complex geometries without the need for expensive tooling and moulds. The use of natural fibres as filament reinforcements was investigated by B. Asaithambi.^84^ Banana/sisal fibres with different lengths (3, 6, and 8 mm) and concentrations were used to fabricate polylactic acid (PLA) filaments, which were subsequently used to fabricate composites *via* fused deposition modelling. They concluded that banana/sisal fibre-reinforced PLA composites may be a promising innovation to improve the performance of these materials, which might enable them to be used in new applications. However, some challenges remain to be solved in the fabrication of the reinforced filaments, such as difficulty in quality control due to voids and porosities, among others.

### 5.2 Morphological characteristics of NFCs

**Fig. 6:**
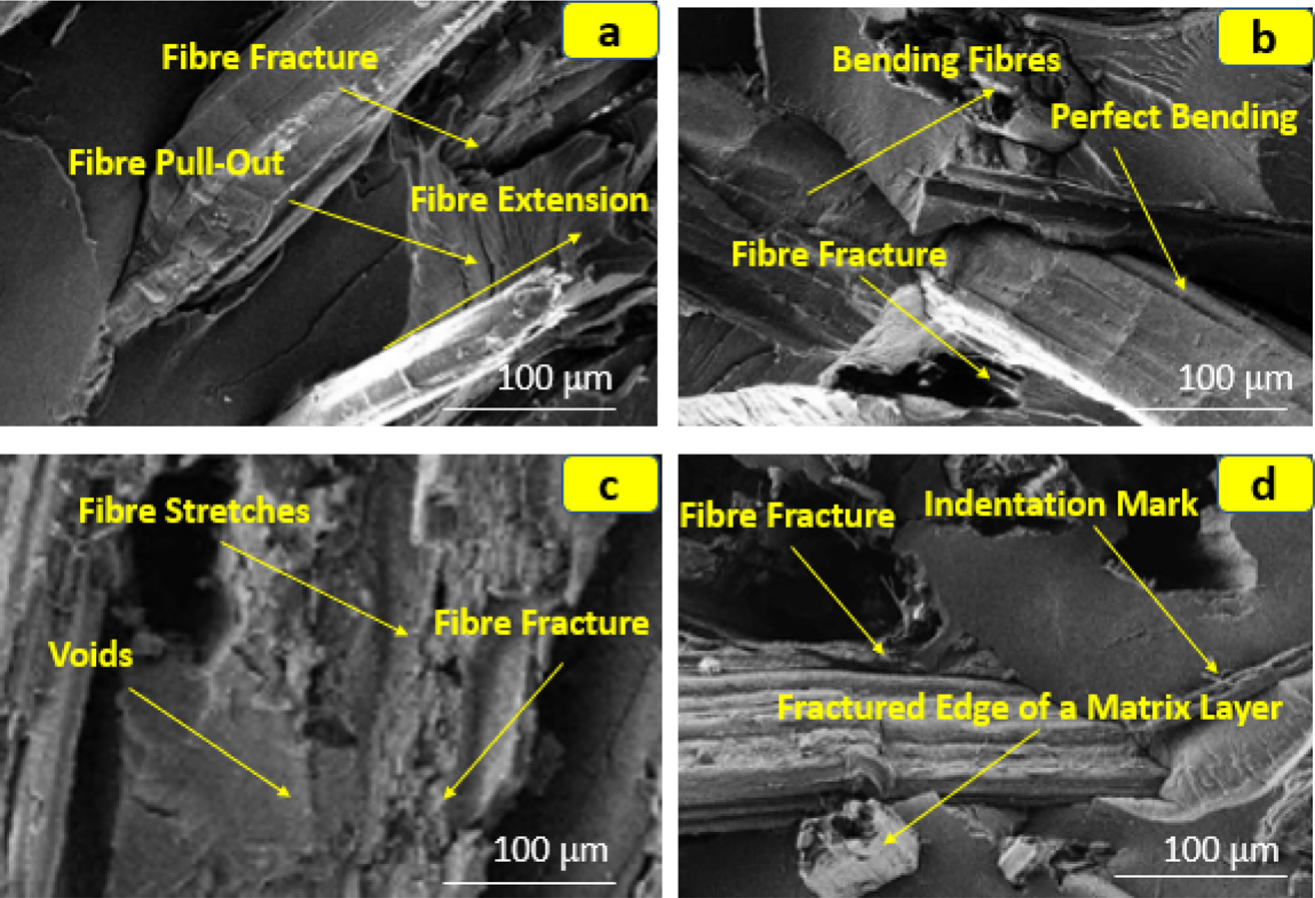
(a–d). SEM images of the fractured specimens of the hemp/kenaf hybrid composites. Adopted from ref. 85 with permission. Copyright MDPI *Journal of Composites Science*, 2023 [Open Access under a Creative Commons License CC BY-NC 4.0 DEED].

**Fig. 7:**
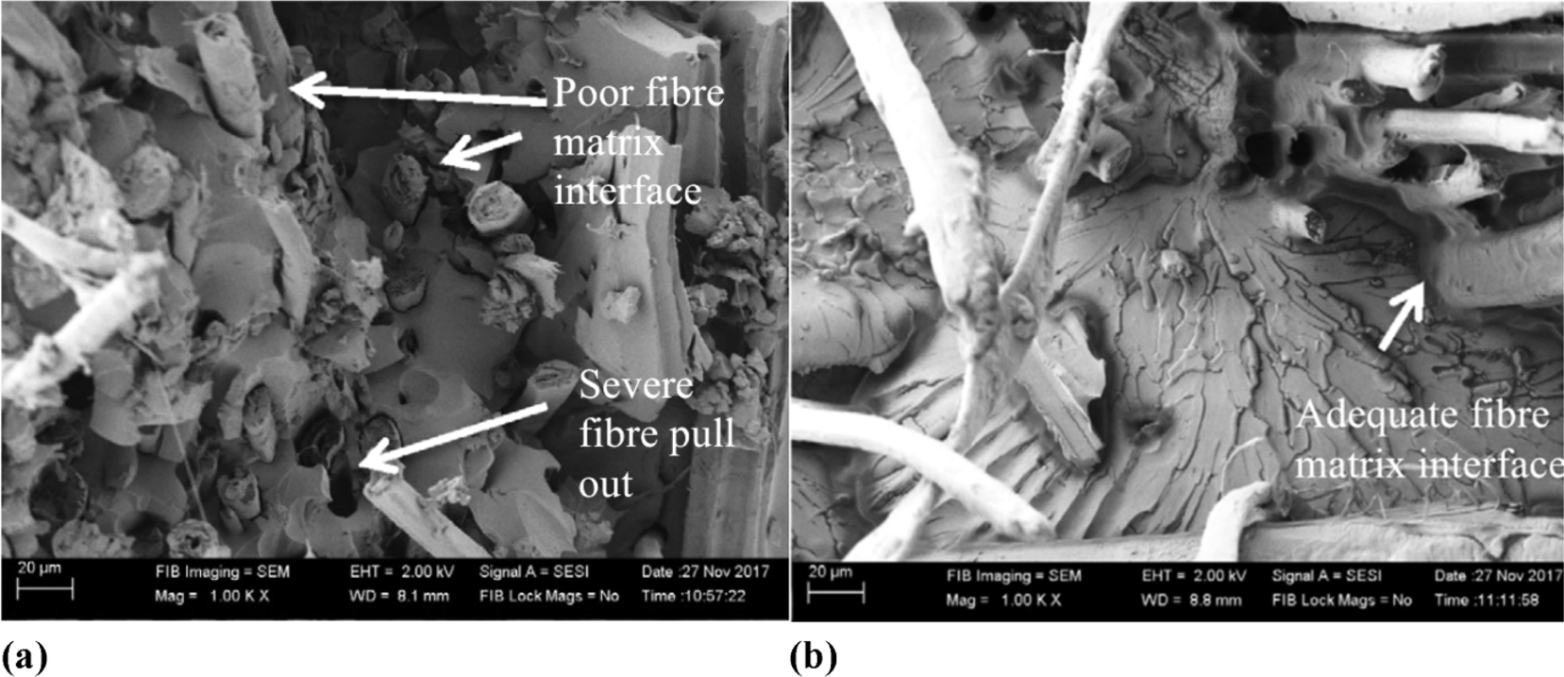
SEM images of the fractured surfaces of a flax-based natural fibre-reinforced composite with (a) 0% TiO_2_ and (b) 0.5% TiO_2_. Adopted from ref. 86 with permission. Copyright Elsevier, 2018.

The morphology at the interfaces of kenaf fibre/hemp fibre/multiwalled carbon nanotube filler with epoxy resin was examined using scanning electron microscopy by Praveena *et al.* in their work. The microstructural features of cracked mechanical surfaces were analyzed. The morphological study encompassed tensile, flexural, and impact fractured specimens, as well as indented test specimens after the hardness test for composites containing kenaf fibre, hemp fibre, and 1% multi-walled carbon nanotubes, as depicted in Fig. 6a–d. This analysis aimed to showcase the improved interaction between epoxy resin and hydroxyl composites. Effective agglomeration of nanoparticles within the dispersed matrix phase was essential. In the third specimen, distinct fibre fractures were observed, alongside prevalent voids due to the matrix layer's fractured edge. The incorporation of hemp fibre/multi-walled carbon nanotubes contributed to enhanced adhesion and interfacial strengthening in the reinforced composite.^85^ Morphological characterization of natural fibre-reinforced composites involves in-depth analysis of material structures at microscopic and nanoscopic scales. The goal is to understand how natural fibres and polymer matrices interact and are distributed within the composite structure. The morphological characterization techniques are commonly used to examine attributes such as fibre-matrix interfaces, dispersion, and crystal orientation. These methods provide crucial insights into composite properties, aiding in understanding performance and design optimization. Fig. 7a and b shows the SEM images of fractured surfaces exhibiting the fibre matrix pull-out and strong bonding between the matrix and the flax fibres with and without TiO_2_ fillers.^86^

### 5.3 Dynamic mechanical analysis, TGA and moisture absorption

The significance of Dynamic Mechanical Analysis (DMA) in evaluating natural fibre-reinforced polymer composites lies in its ability to comprehensively assess their viscoelastic properties, considering factors like temperature, frequency, and applied stress. This technique aids in measuring storage and loss moduli, determining the glass transition temperature, and analyzing viscoelastic behavior, among other parameters crucial for understanding material performance. For instance, a study by Raja *et al.* explored the dynamic mechanical behavior of a novel composite incorporating nanoparticles and natural fibres from banyan and ramie, aiming to enhance mechanical qualities while considering environmental impact. The findings, obtained through DMA analysis, elucidate how the composite's stiffness, energy dissipation, and damping behavior are influenced by the addition of fibres and nanoparticles, offering valuable insights for optimizing the design in industries like automotive and aerospace.^87^ Similarly, thermo-gravimetric analysis (TGA) provides essential information on heat stability and degradation characteristics, as demonstrated by Rajkumar *et al.*'s assessment of pebble filler-based epoxy composites. The study showcases how TGA thermograms reveal the enhanced thermal stability of the composites, indicating potential synergistic interactions between fillers and the matrix.^88^ Additionally, moisture absorption characterization plays a critical role in assessing the resilience of natural fibre composites in damp conditions, with studies like Sanjeevi *et al.*'s investigation shedding light on water absorption kinetics and its implications for mechanical property deterioration. Moisture absorption affects the mechanical characteristics and dimensional stability of NFCs, posing serious problems. When natural fibres are exposed to moisture, they may swell, changing their dimensions, losing strength, and becoming more vulnerable to microbial deterioration. Numerous strategies can be used to address these problems, including reducing the hydrophilicity of fibre surfaces, encapsulating composites, hybridizing with synthetic fibres, changing the polymer matrix with hydrophobic additives, applying sealants or coatings, and designing composite structures to minimize water entrapment. By limiting moisture absorption and reducing its negative impacts, these techniques seek to improve the natural fibre composites' long-term performance and durability in a variety of applications.^89^

## 6. Applications of NFCs

### 6.1 Automotive sector

The automotive industry is keen on leveraging NFCs for benefits like weight reduction, enhanced fuel efficiency, and lowered carbon emissions. These composites find utility in diverse car components such as dashboards, door trims, seat backs, and interior panels. They offer merits including lower density, effective acoustic insulation, vibration damping, and aesthetic appeal, presenting a sustainable alternative to conventional materials in automobiles.

### 6.2 Construction and infrastructure

In construction, NFCs find application across multiple fronts. They reinforce cementitious materials like fibre-reinforced concrete (FRC) to improve crack resistance and flexural strength. Moreover, they are used in roofing materials, wall claddings, insulation panels, and facade elements. Their lightweight nature, thermal insulation prowess, and biodegradability align well with eco-conscious construction practices.

### 6.3 Packaging and consumer goods

The packaging industry is increasingly adopting NFCs for sustainable packaging alternatives. They are employed in crafting biodegradable packaging items such as containers, trays, and disposable cutlery. With attributes like mechanical strength, moisture resistance, and thermal insulation, these composites suit various packaging needs. They combine aesthetic appeal and environmental friendliness in the consumer goods industry, appearing in furniture, toys, home appliances, and kitchenware.

### 6.4 Aerospace and defense

Although their usage is growing, NFCs have a presence in aerospace and defense primarily in non-structural components like cabin linings, seat backs, and interior panels. Offering advantages like weight reduction, fire resistance, and better acoustics, these composites adhere to safety norms. Nonetheless, stringent regulations and the demand for high-performance materials limit their role in crucial aerospace and defense applications.

### 6.5 Other sectors (*e.g.*, sports equipment, furniture)

NFCs have diversified applications, spanning sports equipment and furniture manufacturing. They craft bicycle frames, tennis rackets, helmets, and skateboards, balancing lightweight design with durability and vibration-damping properties. Furniture-wise, they shape chair frames, decorative elements, and table tops, amalgamating natural textures with contemporary designs.

These instances underscore the adaptability and promise of NFCs across industries. Continuous research focuses on refining their traits, exploring novel processing methods, and broadening their role in high-performance contexts. As environmental awareness steers material innovation, the utilization of NFCs is projected to burgeon further.

## 7. Challenges and opportunities

The realm of natural fibre-reinforced polymer composites presents a landscape of challenges and opportunities, as outlined below.

### 7.1 Availability and sourcing of natural fibres

A pivotal hurdle in the broader adoption of NFCs lies in securing appropriate fibre sources. Availability hinges on factors like geography, seasons, and agricultural practices. Sustaining a steady supply of quality fibres with desired attributes is complex. Nonetheless, strides are being made to foster sustainable cultivation methods, harness agricultural waste fibres, and establish robust supply chains. Advancements in biotechnology and genetic engineering also have the potential to elevate fibre quality and availability.^90,91^

### 7.2 Moisture absorption and durability

An inherent trait of natural fibres is their susceptibility to moisture absorption, which can trigger dimensional shifts, mechanical property decline, and eventual degradation. This issue looms large in scenarios where moisture exposure is likely, like outdoor construction materials or automotive parts. Strategies are being explored to temper moisture absorption, including surface treatments, chemical modifications, and infusion of moisture-resistant matrix materials. Augmenting the durability and long-term viability of NFCs is imperative for their effective integration across industries.^92^

### 7.3 Standardization and quality control

Uniformity in performance, aiding material selection and market acceptance, hinges on standardization and quality control. NFCs exhibit variance in fibre attributes, processing techniques, and composite properties. These differences challenge the establishment of consistent testing methods and performance benchmarks. Initiatives are underway to devise standardized testing protocols, material specifications, and quality control procedures specific to NFCs. This stride will empower manufacturers to deliver dependable products, bolstering consumer confidence and market embrace.^93^

### 7.4 Market adoption and cost efficiency

Market acceptability and cost efficiency are pivotal determinants in the widespread integration of NFCs. While interest in sustainable materials is growing, acceptance can be hindered by perceived performance limitations, limited awareness, and preference for conventional options. The cost of NFCs can exceed that of traditional materials due to factors like fibre processing, surface treatment, and quality control expenses. However, through advancements in production techniques, economies of scale, and heightened recognition of environmental advantages, the cost-efficiency of these composites is poised to advance, rendering them more competitive.^94^

Amidst these challenges, NFCs offer promising avenues for sustainable material innovation. Their merits include a small carbon footprint, renewable sourcing, biodegradability, and commendable specific properties. These composites have potential and are in line with the growing emphasis on sustainability and environmental consciousness. NFCs are positioned to replace traditional materials in a variety of industries as research into their performance and solutions continues, ushering in a more sustainable and environmentally friendly future.^95^

## 8. Environmental impact and sustainability

The sustainability and environmental effects of natural fibre-reinforced polymer composites are important considerations in both their creation and use. Several important factors have become apparent and are outlined in the following.

### 8.1 Utilizing renewable resources and lowering carbon footprint

The dependence of NFCs on renewable resources is one of their main advantages. These composites are made from plant-derived fibres that may be grown every year, such as bamboo, hemp, flax, and jute. They are positioned as a strong substitute for non-renewable resources like metals and synthetic polymers because of their renewable nature. Adopting renewable resources promotes a more sustainable material ecology and lessens reliance on finite supplies.^96^

Moreover, composites made of natural fibre have the potential to reduce the carbon footprints of a variety of businesses. Synthetic material production frequently entails energy-intensive processes, chemical synthesis, and significant carbon emissions. Making NFCs, on the other hand, typically requires less energy and produces less carbon emissions. When compared to their synthetic counterparts, natural fibres use less energy to grow and process. Furthermore, a plant's ability to sequester carbon during growth adds to the NFC's total carbon neutrality.

### 8.2 Embracing biodegradability and diverse end-of-life choices

The intrinsic biodegradability of NFCs is an equally important aspect of their sustainability. Natural fibres are biodegradable by nature because they are made of organic components. Consequently, when these composites reach the end of their useful life, they can be disposed of in an environmentally sound manner. Microbial activity drives the biodegradation process, ultimately breaking down the composite into elemental components without leaving behind persistent pollutants or microplastics.^97^

The biodegradability of these composites provides advantages in waste management, alleviating the buildup of non-recyclable materials in landfills. It also fosters prospects for composting and harnessing agricultural waste fibres for other applications. However, ensuring suitable conditions for effective biodegradation, including moisture, temperature, and microbial activity, is paramount for efficient breakdown.^98^

NFC also have end-of-life alternatives beyond biodegradation. They can be recycled or repurposed for secondary applications, curtailing the necessity for raw material extraction and minimizing waste generation. The recycling process entails separating fibres from matrix materials—a complex endeavour given the composite's intricate structure. Nevertheless, progress in recycling technologies and the formulation of composite-specific recycling methods are enhancing the recyclability of NFCs.

The environmental footprint and sustainability of NFCs render them an appealing choice for sectors committed to reducing their environmental impact. By embracing renewable sources, curtailing carbon emissions, and providing biodegradability and versatile end-of-life solutions, NFCs actively contribute to transitioning towards a more sustainable and circular economic framework.^99^ Nonetheless, advancing research and development efforts are pivotal to optimizing the environmental performance of these composites and ensuring their enduring sustainability. Table 2 gives a summary of some of the reviews that have embraced the use of natural fibres for real-time applications.

**Table 2:** Applications of fibres from reviews of the available literature for biodegradability

Fibres	Application of the fibres	Reference
Coir	Container boxes, packaging materials	100
Flax	Panels, decks and dashboards in the automotive industry	101
Hemp	Helmets, bicycles	102
Balsa wood	Modular housing panels	103
Jute	Interior panels of automotive	104
Kenaf	Casing and packaging	105
Wood	Furniture, doors, windows	106

**Fig. 8:**
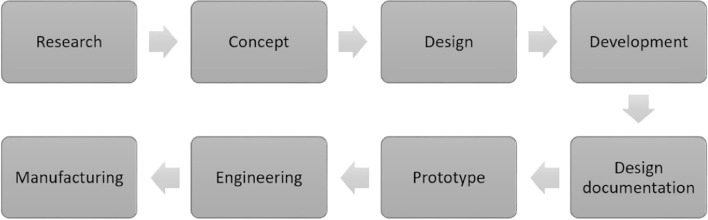
Phases of sustainable product development from Natural fibre-reinforced biocomposites. Adopted from ref. 107 with permission. Copyright MDPI *Polymers* 2021. [Open Access under a Creative Commons License CC BY-NC 4.0 DEED].

The embracing of biodegradability and the subsequent product development for sustainable end-of-life choices depend on the product development phases in the fabrication of the natural fibre-reinforced composites. Fig. 8 shows the flow chart of the product development in the case of the manufacturing of natural fibre-based biocomposites.^107^

The sustainable development of products reinforced with natural fibres involves a comprehensive process encompassing various stages. Initiated by thorough research to address existing gaps in the literature, the outcomes are then translated into conceptual designs, which are further refined through computer simulations, considering real-world conditions and functionality. Prototypes are not fabricated unless these designs are thoroughly documented. Prototypes are put through a rigorous testing process to guarantee their functionality and user safety; this is a crucial validation stage before going into large-scale production. This methodical technique highlights the dedication to sustainability and creativity in the creation of items reinforced with natural fibres.

The use of synthetic polymers as the matrix in NFCs prevents them from complete biodegradation, despite being a more biodegradable option than synthetic composites. This constraint emphasizes how important it is to incorporate biopolymers as a matrix in NFC. Biopolymer resins are a class of polymers derived from renewable natural sources that offer substitutes for traditional petroleum-based plastics. Polylactic acid (PLA) is a popular biodegradable polymer that is sourced from sustainable materials such as sugarcane or maize starch. It has good mechanical qualities and works well with traditional plastic manufacturing techniques. Polyhydroxyalkanoates (PHA) are another class of microorganism-synthesised biodegradable polyesters suitable for a variety of uses, including as medical devices and packaging. Biodegradable alternatives are provided by starch- and cellulose-based polymers obtained from abundant plant sources and can be used in a variety of applications, including biomedical engineering and packaging. Protein-based polymers with unique features that are appropriate for adhesives and biodegradable plastics include zein and soy protein, which are sourced from agricultural sources. PBS, a biodegradable polyester with superior mechanical and thermal stability qualities, is a good choice for packaging and throwaway goods. These biopolymer resins are a prime example of the increasing variety and promise of sustainable materials in resolving environmental issues related to conventional plastics. However, several important factors need to be considered when contrasting synthetic matrices and bio-polymers in composites. Synthetic matrices are frequently used in composite materials because of their well-known resistance to environmental and chemical deterioration. However, because they come from petrochemical sources, they are not biodegradable or renewable, which raises environmental issues. On the other hand, plant-based bio-polymers provide a biodegradable and renewable alternative that complies with environmental goals. Even though bio-polymers have inherent benefits, their mechanical characteristics can require adjustments or reinforcements to bring them up to par with synthetic resins. Bio-polymers provide environmentally suitable substitutes that prioritize biodegradability and renewability, whereas synthetic matrices such as epoxy resins perform well in applications demanding greater endurance and durability in harsh environments. Compared to synthetic matrices, bio-polymers could be more vulnerable to moisture absorption and degradation. Therefore, when using them, several remedial techniques should be considered to decrease water absorption and aging in biopolymer resins to increase their resistance to severe environmental attacks while maintaining their sustainability and biodegradability. One way is to use hydrophobic additives to blend the biopolymer or cross-link it to change its chemical structure. By adding chemical links between polymer chains, cross-linking decreases the number of sites that can absorb water. Hydrophobic additives, such as natural oils or waxes, combine with biopolymers to create a barrier that deters water molecules and reduces absorption. Further limiting water intrusion can be achieved by increasing the density of the polymer and reducing porosity by the optimization of processing parameters like temperature and pressure. Hydrophobic layers can be created on biopolymer surfaces by applying surface treatments like coatings or plasma treatments, which will stop water from penetrating. Adding structural support and narrowing the paths for water molecules to diffuse, and adding fibres or reinforcing fillers to biopolymer matrices can improve their mechanical qualities and reduce water absorption. To put it briefly, aging in biopolymer resins can be efficiently reduced by utilizing a mix of formulation modifications, optimization techniques in processing, and chemical modification. This allows the resins to remain environmentally benign while being suitable for a wide range of applications. The decision between the choice of synthetic matrices and bio-polymers in NFCs ultimately comes down to the demands of the particular application, taking sustainability, performance, and environmental impact into account.

## 9. Comparative analysis of NFCs *versus* conventional materials

Several aspects, including cost-effectiveness, qualities, performance characteristics, and environmental impact, must be considered when comparing NFCs to conventional materials. This is a comparative study that looks at these points.

### 9.1 The effect on the environment

NFCs: natural fibres used in NFCs, such as kenaf, flax, hemp, and jute, are typically sourced from renewable sources. When compared to synthetic fibres used in traditional composites like carbon fibre or fibreglass, they have a lower carbon footprint.

Conventional materials: conventional materials, such as fibreglass or carbon fibre, are produced using energy-intensive extraction and production processes and are obtained from non-renewable resources, such as petroleum. This leads to an increase in greenhouse gas emissions.

### 9.2 Performance attributes

NFCs: natural fibres have excellent specific strength and stiffness, making them appropriate for a range of uses. However, in terms of temperature resistance, impact resistance, and tensile strength, they might not perform as well as synthetic fibres.

Conventional materials: in comparison to natural fibres, conventional materials such as fibreglass and carbon fibre have higher strength-to-weight ratios, superior impact resistance, and the ability to tolerate higher temperatures.

### 9.3 Qualities

NFCs have superior electrical conductivity insulation, are biodegradable, and have strong damping qualities. On the other hand, they might absorb moisture, which could cause dimensional instability and degeneration over time.

Conventional materials: traditional materials, such as carbon fibre and fibreglass, have better mechanical qualities, dimensional stability, and chemical and moisture resistance.

### 9.4 Economy of scale

NFCs: since natural fibres are typically less expensive than synthetic fibres, NFCs have lower material costs. The processing methods and additives needed to improve the characteristics, however, may increase the final cost.

Conventional ingredients: the cost of raw ingredients and production techniques makes conventional materials, such as carbon fibre and fibreglass, relatively expensive. Nonetheless, their increased expense might be justified by their better performance in some applications.

### 9.5 Uses

NFCs are used in consumer goods, packaging, construction materials, and automotive components where environmental factors are crucial.

Conventional materials: high-performance applications requiring excellent mechanical qualities frequently use conventional materials like carbon fibre and fibreglass. These materials are also used in the automotive, sporting goods, and aerospace industries.

In conclusion, NFCs have advantages over conventional materials in terms of cost-effectiveness and environmental sustainability, but their performance may be limited. The needs of the application will determine whether to use NFCs or traditional materials, considering aspects like cost, environmental impact, and performance. Research and development endeavours persist in enhancing the characteristics and competitiveness of NFCs across several industries.

## 10. Enhancing the recyclability of NFC's

Enhancing the NFC's capacity to be recycled in accordance with circular economy tenets is, in fact, an interesting and pertinent subject. Realizing the full potential of NFCs in sustainable material cycles requires developing recycling methods and processes that are specifically suited to them. For these technologies to be adopted and widely accepted, regulatory and certification framework input must be taken into consideration. Standards for NFC recycling procedures can be set by regulatory frameworks, guaranteeing environmental protection and adherence to waste management laws. Certification programs can create market demand and stimulate innovation in recycling technology by assuring consumers and businesses about the sustainability and quality of recycled NFC goods. It is also essential to investigate the social and economic effects of switching to NFCs. Even if there are obvious environmental advantages—like a smaller carbon footprint and a decreased need for non-renewable resources—it is crucial to comprehend the wider effects on businesses, communities, and economies. Making the switch to NFCs might upend established industries dependent on conventional materials and open new employment prospects in the recycling and sustainable manufacturing sectors. Furthermore, evaluating the social equity components—such as the price and accessibility of NFC-based products—ensures that the shift is equitable and advantageous to all societal groups. All things considered, talking about legislative frameworks and taking social and economic effects into account enhances the conversation about improving NFCs' recyclability and moving closer to a circular economy.

The evolution of NFCs and their recyclability have offered prospects related to the use of NFCs for industry practitioners with valuable insights as compared to conventional materials. One prominent example of the use of NFC replacing conventional materials is in the automotive sector, where NFCs are gradually taking the place of traditional materials like carbon fibre and fibreglass. Companies such as Toyota and BMW, for example, have incorporated NFCs into dashboards, seat backs, and door panels among other car components to minimize weight and increase fuel efficiency without sacrificing mechanical qualities.

Researchers at the University of Stuttgart assessed the performance of NFCs derived from flax fibres in automobile applications. The results of the study showed that flax-based NFCs had mechanical qualities that were on par with those of traditional materials, like glass fibre-reinforced composites, but with less of an adverse effect on the environment, and biodegradability. Nonetheless, issues with dimensional stability and moisture absorption were noted, emphasizing the need for more study and development to maximize NFCs for use in automobiles.^108^

Another interesting example is the construction sector, where NFCs are being investigated more and more as environmentally friendly substitutes for traditional building materials like concrete and steel. A study was carried out by researchers at the University of Bath on the application of NFCs in building construction structural elements like beams and columns. According to the study, bamboo fibre-based NFCs had favourable mechanical qualities, such as a high strength-to-weight ratio and flexibility, which qualified them for use in load-bearing applications. However, difficulties with durability and fire resistance in outdoor settings were found, highlighting the significance of resolving these problems to enable broader use of NFCs in buildings.^109^

Through the practical instances mentioned here, scholars and professionals in the field can gain significant understanding regarding the capabilities, advantages, and difficulties of NFCs in contrast to traditional materials. Future studies and technical developments aiming at improving NFCs' performance, sustainability, and ability to be recycled can benefit from these insights, which will ultimately increase the acceptance of NFCs across a range of industries and aid in the shift to a circular economy.

## 11. Conclusions

This review explores the field of NFCs and how material engineering might advance sustainability through its use. Several important conclusions have come from the review:

• NFCs offer a sustainable alternative to traditional materials, exhibiting benefits such as reduced carbon emissions, biodegradability, and renewable supply.

• Plant-derived fibres, such as jute, hemp, and flax, are widely used in these composites because of their availability, mechanical properties, and eco-friendliness.

• To achieve the desired composite characteristics and performance, critical processing processes that include fibre extraction, preparation, modification, and matrix formulation are essential.

• Different reinforcement methods—from fibres oriented randomly to equivalents that are aligned and hybrid composites—allow for the enhancement of mechanical characteristics to meet the needs of certain applications.

• Fibre type, fibre/matrix interaction, and processing techniques all affect the mechanical properties of these composites, including tensile and flexural strength, impact resistance, thermal conductivity, and water absorption.

• NFCs are popular in many areas, such as packaging, construction, automotive, and aerospace. Benefits from its applications include reduced weight, improved acoustic insulation, and compliance with environmental goals.

## 12. Scope of future work

The NFCs have great promise for sustainable material engineering, however, there are a few areas that still need to be explored and researched further.

• Improving the mechanical properties of NFCs and improving their performance in demanding applications. This means developing new reinforcement techniques, optimizing adhesion at the fibre/matrix interface, and creating sophisticated processing schemes.

• Setting up established processes for standardization and quality assurance that are specific to NFCs is crucial. These kinds of steps are essential to guarantee material performance consistency and promote broader market adoption.

• More thorough life cycle assessments (LCAs) that cover the extraction, processing, use, and disposal of raw materials, as well as the overall environmental effect of NFCs, are desperately needed.

• Enhancing the recyclability of NFCs and aligning them with the ideas of a circular economy requires the development of recycling technologies and procedures that are specific to composites.

• To propel the progress in NFCs, fruitful collaboration among academia, industry, and governmental bodies is indispensable. This synergy drives research, development, and eventual commercialization endeavours.

• The dissemination of research findings pertaining to the evolution of novel product iterations stemming from natural fibre-reinforced composites is essential. This effort serves to illuminate the advantages and applications of these composites among designers, engineers, and consumers alike.

By addressing these forthcoming trajectories and research imperatives, the adoption of NFCs can be expedited, ushering in a more sustainable and ecologically mindful approach to material engineering. Continual exploration and enhancement of these materials will undoubtedly contribute to the realization of a greener and more sustainable future.

## Consent for publication

All authors consent to the publication of this manuscript.

## Data availability

The necessary data used in the manuscript are already present in the manuscript.

## Author contributions

All authors listed have significantly contributed to the development and the writing of this article.

## Conflicts of interest

The authors declare no conflict of interest.

## Supplementary Material
